# Optimizing Timing of Immunotherapy Improves Control of Tumors by Hypofractionated Radiation Therapy

**DOI:** 10.1371/journal.pone.0157164

**Published:** 2016-06-09

**Authors:** Kristina H. Young, Jason R. Baird, Talicia Savage, Benjamin Cottam, David Friedman, Shelly Bambina, David J. Messenheimer, Bernard Fox, Pippa Newell, Keith S. Bahjat, Michael J. Gough, Marka R. Crittenden

**Affiliations:** 1 Earle A. Chiles Research Institute, Robert W. Franz Cancer Center, Providence Portland Medical Center, 4805 NE Glisan St, Portland, OR, 97213, United States of America; 2 The Oregon Clinic, Portland, OR, 97213, United States of America; 3 Providence Hepatobiliary and Pancreatic Cancer Program, Providence Portland Medical Center, 4805, NE Glisan St, Portland, OR, 97213, United States of America; Istituto Superiore di Sanità, ITALY

## Abstract

The anecdotal reports of promising results seen with immunotherapy and radiation in advanced malignancies have prompted several trials combining immunotherapy and radiation. However, the ideal timing of immunotherapy with radiation has not been clarified. Tumor bearing mice were treated with 20Gy radiation delivered only to the tumor combined with either anti-CTLA4 antibody or anti-OX40 agonist antibody. Immunotherapy was delivered at a single timepoint around radiation. Surprisingly, the optimal timing of these therapies varied. Anti-CTLA4 was most effective when given prior to radiation therapy, in part due to regulatory T cell depletion. Administration of anti-OX40 agonist antibody was optimal when delivered one day following radiation during the post-radiation window of increased antigen presentation. Combination treatment of anti-CTLA4, radiation, and anti-OX40 using the ideal timing in a transplanted spontaneous mammary tumor model demonstrated tumor cures. These data demonstrate that the combination of immunotherapy and radiation results in improved therapeutic efficacy, and that the ideal timing of administration with radiation is dependent on the mechanism of action of the immunotherapy utilized.

## Introduction

Radiation therapy influences the patient’s immune system and the immune system influences the response to radiation therapy. [1] Radiation therapy of tumors results in a dose-related increase in MHC class I expression [2] and a short window of antigen presentation within 2 days following high-dose radiation. [3] Many of the preclinical and clinical immune therapies targeting T cells thus apply costimulation or immune adjuvants closely following doses of radiation. [4–8] These approaches have been shown to varying degrees to increase tumor-antigen specific immune responses, improve clearance of radiation treated and distant untreated tumors, and protect cured animals from subsequent tumor challenge. However, a series of interesting anecdotal reports have demonstrated that immune therapy with ipilimumab (human anti-CTLA4 antibody) followed by radiation can lead to extensive tumor regression with increased tumor antigen specific responses. [9, 10] In these patients, radiation therapy was delivered in a palliative manner to individual lesions in patients already participating in Ipilimumab studies. Ipilimumab therapy has been shown to increase T cell infiltrates into tumors in patients, regardless of whether these tumors exhibit a response to antibody therapy. [11] Thus, those patients who achieved both local and distant disease control with focal palliative radiation delivered following immune therapy would likely have received treatment to an immunologically more favorable tumor immune environment. In a review of patients treated with ipilimumab and radiation, patients treated with radiation following immune therapy, in the ‘maintenance phase’, showed a significant survival advantage over those treated with radiation during the ‘induction phase’. [12] These data suggest the efficacy of anti-CTLA4 and radiation therapy can be improved by optimizing timing.

To date, few studies have attempted to optimize the timing of immunotherapy with radiation such that immunotherapy is delivered first. We recently demonstrated in preclinical murine models of radiation therapy that pre-treatment with TGFβ inhibitors improved the response to radiation therapy by improving immune control of residual disease. [13] We hypothesize that depending on the mechanism of action of immunotherapy that the optimal timing of radiation and immunotherapy will be different. This is important to identify as currently, the majority of clinical trial designs which deliver anti-CTLA4 therapy concurrent with or following radiation do not take into account anecdotal reports suggesting that palliative radiation delivered to patients undergoing anti-CTLA4 therapy resulted in systemic therapeutic responses. [9, 10] In this study, we test the optimal timing of two distinct immunotherapy approaches, a checkpoint inhibitor and a co-stimulatory agonist, when combined with radiation. We demonstrate that pre-treatment with anti-CTLA4 antibodies provided optimal tumor control, while an alternate immunotherapy with anti-OX40, which targets recently-activated T cells, was optimal if delivered immediately following radiation therapy. We demonstrate that the efficacy of anti-CTLA4 pretreatment may lie in its ability to delete regulatory T cells. This study provides important preclinical evidence to consider when translating combinatorial treatment to the clinic, specifically allowing a tailored approach that takes into account the immunotherapy mechanism of action when planning the optimal timing of radiation.

## Methods and Materials

### Animals and cell lines

The CT26 murine colorectal carcinoma [14] was obtained from ATCC (Manassas, VA). Cells were grown in RPMI-1640 media supplemented with HEPES, non-essential amino acids, sodium pyruvate, glutamine, 10% FBS, penicillin and streptomycin. All cell lines tested negative for mycoplasma. BALB/c and FVB mice were obtained from Jackson Laboratories (Bar Harbor, ME). FVB mice bearing the MMTV-PyMT transgene [15] were kindly provided by Dr. Akporiaye (EACRI, Portland OR) and heterozygous PyMT^+^ mice that spontaneously develop mammary tumors and PyMT^-^ tumor-free littermates were recruited into comparative studies. Tumor bearing mice were monitored a minimum of three days per week and euthanized when tumors exceeded 12mm in any dimension, or when body condition score declined one level. Euthanasia was performed with CO2 inhalation followed by a second method, either organ harvest or cervical dislocation. Radiation was performed with inhaled isoflurane anesthesia, intraperitoneal meloxicam was given for analgesia. There were no unexpected animal deaths. All animal protocols were approved by the Earle A. Chiles Research Institute IACUC (Animal Welfare Assurance No. A3913-01).

### Antibodies and reagents

Fluorescently-conjugated antibodies CD3-e450, CD8-PerCP, CD4-FITC, CD4-e450, CD4-PerCP, CD25-APC, were purchased from Ebioscience (San Diego, CA). CD8-PE-TxRD was purchased from Invitrogen (Carlsbad, CA). Therapeutic anti-CTLA4 (clone 9D9 or UC10), anti-OX40 (clone OX86), anti-CD40 (clone FGK4.5), anti-CD4 (clone GK1.5), and anti-CD25 (clone PC.61.5.3) antibodies were obtained from BioXcell (Branford, CT) and resuspended in sterile PBS to a concentration of 1mg/mL. Antibodies were administered as 250μg (anti-OX40 and anti-CTLA4) or 100μg (anti-CD4 and anti-CD25) intraperitoneally. DEC205ova was kindly provided by CellDex Therapeutics (Hampton, NJ). SIINFEKL-Kb tetramers were obtained from the NIH Tetramer Core Facility at Emory University (Atlanta, GA).

### In Vivo Radiotherapy Models

1x10^4^ CT26 cells were injected in 100μL of PBS subcutaneously in the right hind limb of immunocompetent BALB/c mice. Radiation was delivered using the clinical linear accelerator (6MV photons, Elekta Synergy linear accelerator, Atlanta, GA) with a half-beam block to protect vital organs and 1.0cm bolus to increase the dose to the tumor. 20Gy x 1 was delivered on day 14 [13]. For mice cured of CT26 tumors, mice were rechallenged with 5x10^4^ 4T1 and 1x10^4^ CT26 tumors in opposite flanks to assess tumor-specific immunity. Radiation was performed on the clinical linear accelerator prior to acquisition of the Small Animal Radiation Research Platform (SARRP) described below.

For therapeutic studies, MMTV-PyMT tumors were established in naïve FVB mammary glands using the published method [16]. Briefly, tumors were harvested from day 100 MMTV-PyMT^+^ mice and dissected into approximately 2mm fragments followed by agitation in 1mg/mL collagenase in PBS for 1hr at room temperature. The digest was filtered through 100μm nylon mesh to remove macroscopic debris and 1x10^6^ cells were injected into the mammary fat pat in a 1:1 mix with Matrigel (BD Biosciences, Franklin Lakes, NJ). For radiation therapy of these tumors, mice were anesthetized by isolflourane inhalation on the stage of a Small Animal Radiation Research Platform (SARRP, XStrahl, GA), and CT imaged. Dosimetry was performed using SLICER software with SARRP-specific add-ons (XStrahl) and treatment calculated to an isocenter in the tumor target. Treatment plans used doses split between two opposing beams with a 10mm collimator delivered at a tangent to minimize dose to the torso. SARRP treatments were necessary to target the orthotopic mammary tumor and minimize dose to the torso, which would not have been feasible using the clinical linear accelerator.

### Flow cytometry

For analysis of cell depletion or antigen-specific cell numbers in blood, whole blood was harvested into EDTA tubes from live mice via the saphenous vein, and fresh blood was stained directly with fluorescent antibody cocktails along with Kb-SIINFEKL tetramers where appropriate. Red blood cells were lysed with Cal-Lyse whole blood lysing solution (Invitrogen), and samples analyzed on a BD LSRII flow cytometer.

### Statistics

Data were analyzed and graphed using Prism (GraphPad Software, La Jolla, CA). Individual data sets were compared using Student’s T-test and analysis across multiple groups was performed using ANOVA with individual groups assessed using Tukey’s comparison. Kaplan and Meier survival curves were compared using a log-rank test.

## Results

**Fig 1:**
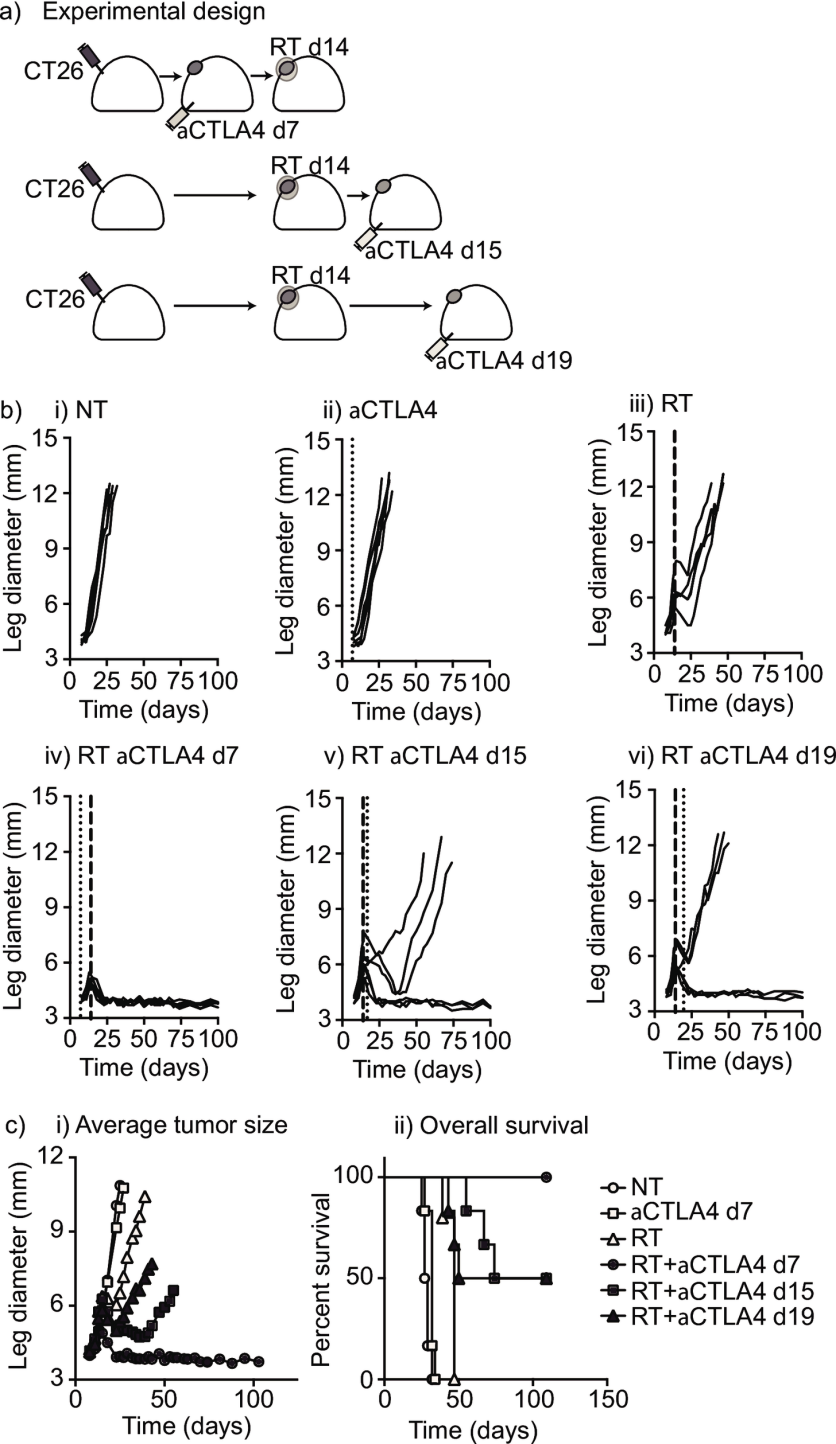
Anti-CTLA4 pretreatment optimizes tumor control by radiation therapy. (a) Immunocompetent BALB/c mice bearing CT26 tumors were left untreated (NT) or treated with 250μg anti-CTLA4 on d7, d15 or d19. Select groups were additionally treated with 20Gy focal radiation (RT) on d14. (b) Individual tumor measurements from mice in these groups i) untreated or treated with ii) anti-CTLA4 d7; iii) RT 20Gy d14; iv) anti-CTLA4 d7+RT 20Gy d14; v) anti-CTLA4 d15+RT 20Gy d14; vi) anti-CTLA4 d19+RT 20Gy d14. (c) i) average tumor size and ii) overall survival. Representative experiment shown with n = 6 mice per group. Experiment replicated a minimum of two times.

Increasingly immunotherapy is combined with radiation to enhance response; however, relatively little data exist regarding the ideal timing of combination therapy. Currently, the majority of clinical trial designs which deliver anti-CTLA4 therapy concurrent with or following radiation, do not take into account anecdotal reports suggesting that palliative radiation delivered to patients undergoing anti-CTLA4 therapy resulted in systemic therapeutic responses [9, 10]. Therefore, we investigated the effect of anti-CTLA4 immunotherapy timing with regard to radiation. We established CT26 colorectal tumors in the right hindlimb of syngeneic BALB/c mice, and treated mice with anti-CTLA4 antibody on either day 7, day 15, or day 19; 20Gy radiation was delivered to the tumor only, on day 14 (**Fig 1A**). A single dose of radiation was chosen to simplify the timing of immune therapy relative to radiation, while 20Gy was chosen given the *in vivo* radioresponse of CT26 tumors^13^ and data demonstrating dose-dependent increase in MHC I expression and antigen presentation.[2]^,^[3] Anti-CTLA4 treatment alone had little effect on tumor growth (**Fig 1B**) and resulted in a small survival benefit with a median survival of 32 days versus 28 days in the no treatment (NT) control group (p = 0.03) (**Fig 1C**). While radiation alone resulted in transient tumor control (**Fig 1B**), all tumors regrew resulting in euthanization secondary to tumor burden with a median survival of 47 days (p = 0.0014 versus NT) (**Fig 1C**). Tumor-bearing mice that received anti-CTLA4 on day 7 prior to radiation cleared their tumors with an undefined median survival (p = 0.002 vs radiation alone) (**Fig 1B and 1C**). The mean tumor size of mice pretreated with anti-CTLA4 versus control mice was not significantly different at the time of radiation therapy. Half the tumor-bearing mice that received anti-CTLA4 following radiation cleared the tumor with median survivals of 92 days for day 15 administration (p = 0.002 vs radiation alone) versus 53 days for day 19 administration (p = 0.07 vs radiation alone) (**Fig 1B and 1C**). Importantly, all mice cured of tumors by combination therapy were resistant to rechallenge with CT26 tumors, but remained susceptible to a different tumor, indicating long-term tumor-specific immunity was achieved (**Table 1**). These data demonstrate that the addition of anti-CTLA4 to radiation therapy improves survival regardless of timing, but is most effective when delivered before radiation.

**Table 1:** Tumor-bearing mice cured of CT26 tumors were rechallenged after 100 days with CT26 and 4T1 on opposing flanks. Resulting tumor growth demonstrated all mice cured of CT26 rejected rechallenge with CT26, but succumbed to syngeneic, but immunologically distinct 4T1 tumors.

CT26 primary tumor	Tumors from rechallenge with:	
	CT26	4T1
Anti-CLTA4 + RT	0/17	17/17
Anti-OX40 + RT	0/13	13/13
RT alone	0/3	3/3

**Fig 2:**
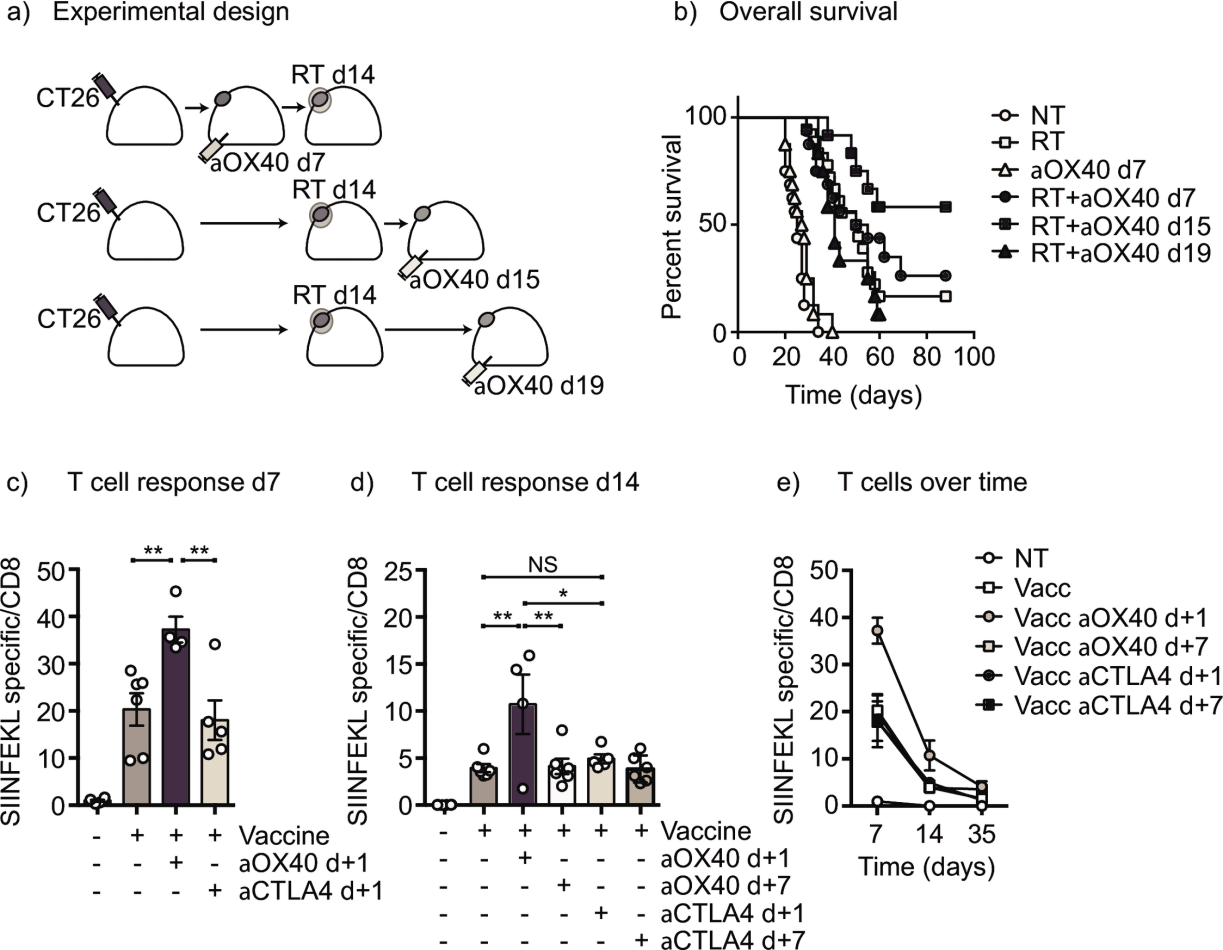
Timing of immunotherapy is dependent on the mechanism of action. (a) Immunocompetent BALB/c mice were challenged with CT26 in the flank and randomized to no further treatment (NT), 20Gy focal radiation therapy (RT) alone or along with administration of 250μg anti-OX40 on d7, d15 or d19. (b) Overall survival of mice following treatment. Data combined from 3 experiments, total n = 12–18 mice per group. (c) Immunocompetent C56BL/6 mice were left untreated (NT) or vaccinated with DEC205ova plus anti-CD40 (Vaccine) on d0. Matched groups of vaccinated mice were additionally treated with 250μg anti-OX40 on d1 or d7, or 250μg anti-CTLA4 on d1 or d7. Graph shows the percent of CD8 T cells that are specific for the immunodominant SIINFEKL peptide of Ova on (c) d7, (d) d14 or (e) over time. Key: NS = not significant; * = p<0.05; ** = p< 0.01. Each symbol represents one animal.

To determine whether immunotherapy preceding radiation is always the optimal sequencing of these two modalities, we evaluated the effect of timing on the efficacy of anti-OX40 immunotherapy, a co-stimulatory antibody rather than a checkpoint inhibitor, with radiation. OX40 (CD134) is a member of the TNFR superfamily and, unlike CTLA4, OX40 is transiently induced on T cells immediately following antigen exposure [17]. We and others have previously demonstrated that delivery of an agonist antibody to OX40 immediately following radiation therapy significantly increased survival in the 3LL lung carcinoma model [5, 18], but it is possible that this timing is also not optimal. We again established CT26 colorectal tumors in the hindlimb of BALB/c mice and delivered an anti-OX40 agonist antibody on day 7, day 15, or day 19; 20Gy radiation was delivered to the tumor only on day 14 (**Fig 2A**). Contrary to what we observed with anti-CTLA4 therapy in combination with radiation, pretreatment with anti-OX40 antibodies did not provide any therapeutic advantage over radiation alone (median survival 55 days versus 48 days, p = 0.23) (**Fig 2B**). Delayed anti-OX40 administration at day 19, also did not provide a benefit over radiation alone (median survival 41 days, p = 0.6). However, anti-OX40 delivered one day following radiation resulted in ~50% of mice clearing their tumors (116.5 days, p = 0.0006 vs radiation alone) (**Fig 2B**). This result agrees with prior studies demonstrating that anti-OX40 must be present during the key period, 12–24 hours following antigen exposure to coincide with OX40 upregulation on T cells [17], and with the evidence that tumor antigen-presentation approximately 2 days following radiation therapy [3], suggesting that 5 days post-radiation therapy will be beyond this therapeutic window. Importantly, all mice cured of tumors by optimal timing of anti-OX40 were resistant to rechallenge with CT26 tumors, but remained susceptible to a syngeneic antigenically distinct tumor, indicating long term antigen-specific immunity was achieved (**Table 1**).

We propose that anti-OX40 functions to boost antigen specific T cell numbers whereas anti-CTLA-4 would not. Therefore anti-OX40 would need to be present in close conjunction to antigen release. To compare the ability of anti-OX40 versus anti-CTLA4 to boost antigen-specific immune responses, non-tumor bearing normal mice were left untreated or vaccinated with Dec205-ova plus anti-CD40. This generates a strong antigen-specific immune response as measured by antigen-specific recognition of SIINFEKL-tetramers in the peripheral blood 7 days following vaccination (**Fig 2C–2E**). The addition of anti-OX40 antibodies 1 day following antigen significantly increased the proportion of antigen-specific T cells, however the addition of anti-CTLA4 antibodies did not change the CD8 T cell response (**Fig 2C**). Addition of anti-OX40 antibodies 7 days following vaccination did not increase antigen-specific T cell numbers, consistent with loss of OX40 expression (**Fig 2D**). Anti-CTLA4 antibodies failed to cause T cell expansion when administered 7 days following antigen. SINFEKL specific T cells, while significantly greater in the anti-OX40 d+1 treated animals, decreased in proportion over time in all groups, returning to non-significant levels by day 35 (**Fig 2E**). These data confirm that anti-OX40 but not CTLA-4 enhances antigen specific T cells and suggests that this agonist antibody needs to be present when antigen presentation is occurring.

**Fig 3:**
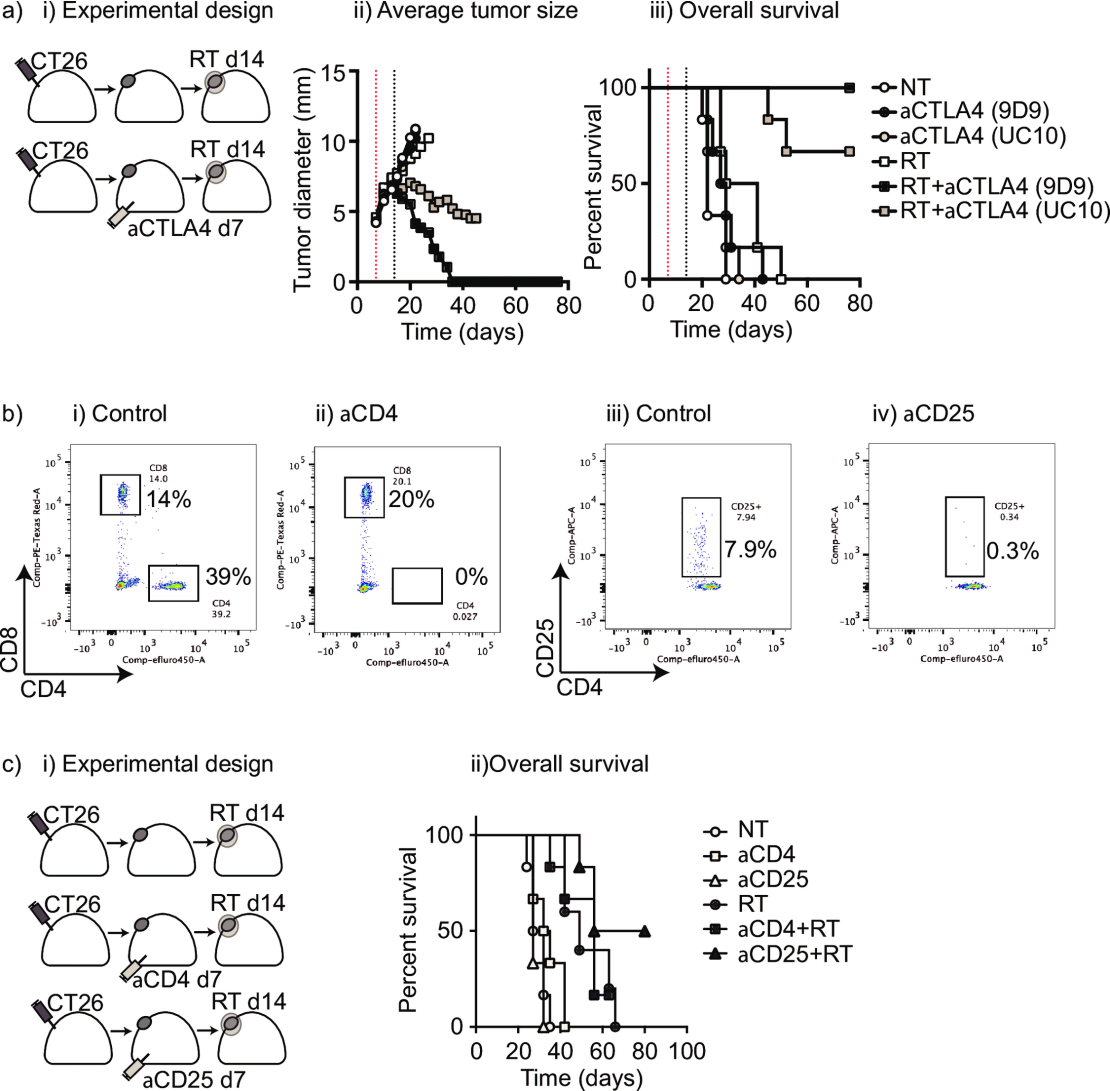
**Effect of Treg depetion on tumor control by radiation therapy** (a) i) Immunocompetent BALB/c mice bearing CT26 tumors were left untreated (NT) or treated with 250μg anti-CTLA4 clone 9D9 or UC10 on d7. Select groups were additionally treated with 20Gy focal radiation (RT) on d14. Graphs show ii) average tumor size and iii) overall survival. (b) Immunocompetent BALB/c mice bearing CT26 tumors were left i+iii) untreated (control) or treated with ii) 100μg anti-CD4 depleting antibody or iv) anti-CD25 depleting antibody. Representative flow cytometry plots show whole blood 1 day following control or antibody-depletion i+ii) gated on lymphocytes and stained for CD8 and CD4 or iii) and iv) gated on CD4^+^ lymphocytes and stained for CD4 and CD25. (c) i) Immunocompetent BALB/c mice bearing CT26 tumors were left untreated (NT) or treated with 100μg anti-CD4 or anti-CD25 on d7. Select groups were additionally treated with 20Gy focal radiation (RT) on d14. ii) Overall survival in all groups.

To understand how anti-CTLA4 pretreatment enhances tumor control by radiation therapy, we investigated the mechanism of action of this checkpoint inhibitor. Recent reports demonstrate that anti-CTLA4 antibodies cause Fc-dependent depletion of regulatory T cells in the tumor [19, 20] and it has been shown that depletion of regulatory T cells concurrent or following radiation therapy resulted in enhanced tumor control.[21, 22] Since different anti-CTLA4 clones have been shown to differ in depletion of regulatory T cells, we tested the following different clones in combination with radiation therapy: the 9D9 clone that is highly depleting, and the UC10 clone which is less depleting.[19] As before, we established CT26 tumors in the hindlimb of immunocompetent Balb/c mice and administered either the 9D9 or the UC10 clone on day 7 followed by radiation on day 14 (**Fig 3Ai**). While all mice treated with 9D9 and radiation cleared their tumors, only 67% of mice treated with the UC10 clone cleared their tumors (**Fig 3Aii and 3Aiii**). To determine whether the improved radiation efficacy of anti-CTLA4 prior to radiation could be fully explained by regulatory T cell depletion, we established CT26 tumors in the hindlimb of BALB/c mice and treated on day 7 with anti-CD4 to deplete all CD4 T cells or anti-CD25 to deplete T regulatory cells. Mice were treated with radiation therapy on day 14 as above. Antibody treatment efficiently depleted CD4^+^ or CD25^+^ cells in the mouse (**Fig 3B**). CD4 depletion alone or in combination with subsequent radiation therapy did not affect tumor growth (**Fig 3C**). CD25 depletion alone did not affect tumor growth, but when followed by radiation therapy resulted in cure of tumors in half of the mice. Importantly, CD25 depletion did not perform as well as our prior studies with anti-CTLA4 pretreatment, and total CD4 depletion, which would include both regulatory and effector T cell depletion, was not effective. From this we hypothesize that anti-CTLA4 is providing effects in addition to regulatory T cell depletion, and that non-regulatory CD4 cells must be required for the cures in CD25-depleted animals. These results may be confounded by the fact that we have previously demonstrated that increased proportions of antigen-responsive CD8^+^CD25^+^ cells repopulate tumors following radiation therapy [5], and these cells may also be depleted by anti-CD25 treatment. Nevertheless, it is likely that anti-CTLA4 therapy plays a dual role by both removing pre-existing T regulatory cells and by blocking CTLA4-mediated suppression of CD4 and CD8 effector T cells; together permitting improved clearance of residual cancer cells following radiation therapy.

**Fig 4:**
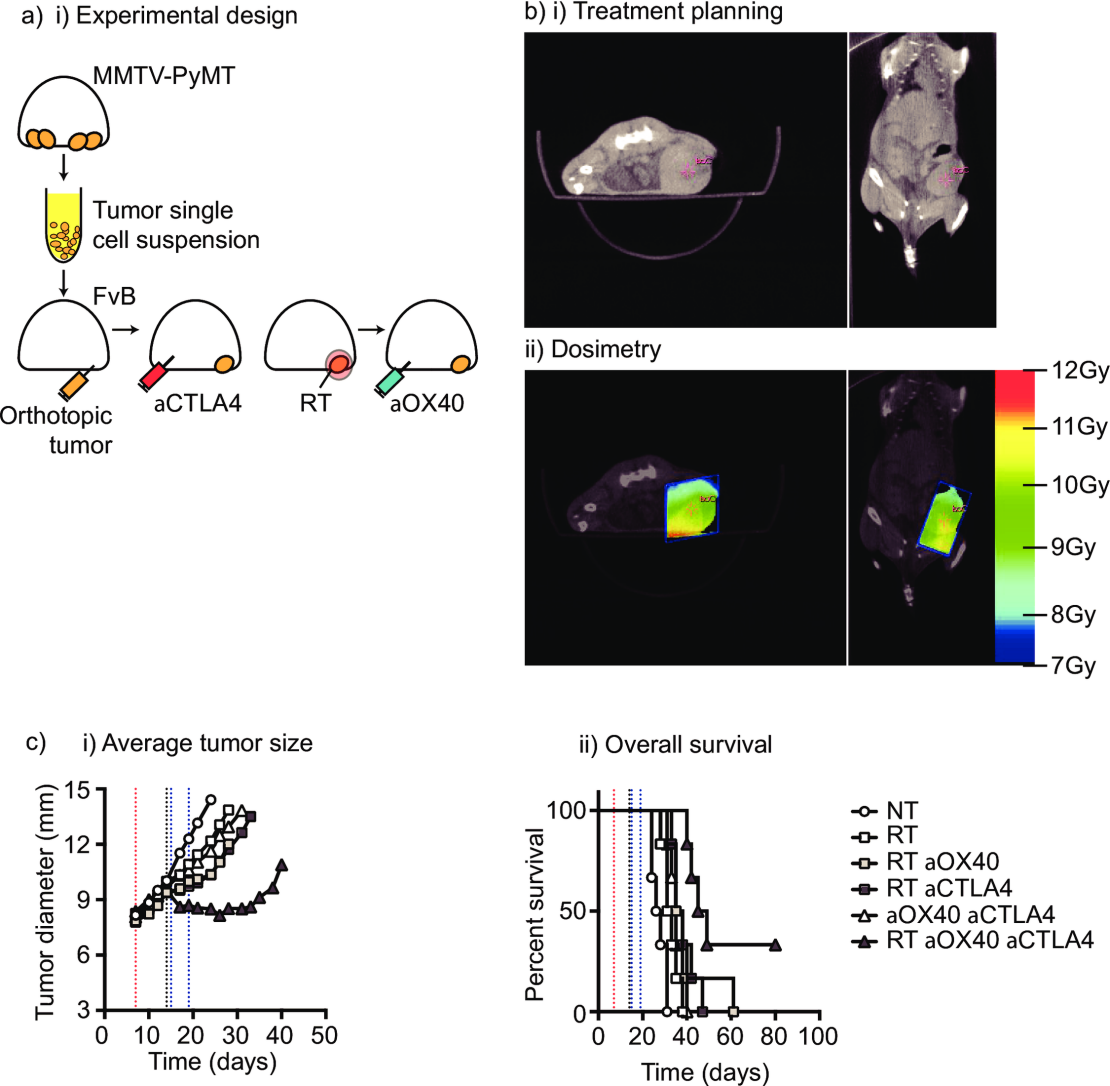
Combination immunotherapy and radiation therapy of spontaneous mammary tumors in immune competent mice. (a) MMTV-PyMT tumors were harvested from approximately 100 day old female MMTV-PyMT^+^ mice, the tumor disrupted ex vivo and 1x10^6^ viable cells injected orthotopically into immunocompetent syngeneic FVB mice. (b) Mice with d14-d17 tumors underwent CT-guided radiation therapy (RT) using a Small Animal Radiation Research Platform and i) images used to place isocenters within individual mammary tumors and collimeters and beam angles designed to deliver focal radiation to the tumor and minimal dose to radiosensitive organs. ii) CT images were segmented by tissue density and this information used to predict dose delivery. Mice were also randomized to receive 250μg anti-CTLA4 immunotherapy 7d prior to RT, and 250μg anti-OX40 immunotherapy d1 and d4 following radiation. (c) Graphs show i) average tumor growth and ii) overall survival.

Transplantable tumors can carry very large mutational loads due to their mode of initiation and their extended time in culture, and the CT26 tumor is a particularly immunogenic tumor model. While unresponsive to immunotherapy alone, it is highly responsive to immunotherapy combined with radiation therapy. By contrast, many tumors in patients have lower mutational burden and appear to be more resistant to monotherapy with individual checkpoint inhibitors. To study whether combinatorial therapies delivered at optimal immunotherapy timing can still impact tumors with lower mutational burden we aimed to develop a model of radiation therapy in mice bearing spontaneous tumors. These tumors would be anticipated to exhibit a significantly lower mutational burden [23]. To test for survival advantage we used a previously described model where spontaneous MMTV-PyMT tumors are transplanted into the mammary fat pads of naïve FVB mice [16] (**Fig 4A**). These mammary tumors were allowed to develop for 14 days then left untreated or treated with a single 10Gy dose of focal radiation using CT guidance to permit radiation therapy of the orthotopic tumor with minimal dose to normal structures (**Fig 4B**). Immunotherapy with anti-OX40 has been shown to synergize well with anti-CTLA4 [24], therefore we tested the effect of combining anti-CTLA4 at its optimum timing pre-radiation with anti-OX40 post-radiation. Mice were randomized to receive anti-CTLA4 day 7 post-implantation and with anti-OX40 therapy on days 15 and 18, consistent with the ideal timing determined in CT26 tumors, but with an additional dose given the expected therapeutic resistance of this model. As we have seen in other tumor models [5, 25, 26], radiation alone resulted in transient tumor control followed by aggressive outgrowth and an increase in median survival from 26 to 36 days (p<0.001 **Fig 4C**). The combination of radiation therapy and anti-OX40 therapy significantly extended survival compared to anti-OX40 alone (p<0.001) but this was only significantly greater than radiation therapy alone in 2 of 3 repeats of this experiment.[27, 28] Combination treatment with anti-CTLA4, anti-OX40 and radiation resulted in significantly extended survival compared to anti-CTLA4+anti-OX40 (p<0.001), RT alone (p<0.01), RT+anti-OX40 (p<0.05) and RT+anti-CTLA4 (p<0.05) (**Fig 4C**). These data demonstrate that in aggressive spontaneous mammary carcinomas, radiation therapy combined with immunotherapy delivered at its optimum timing can significantly extend survival and importantly can result in tumor cures.

## Discussion

Over the past several years there has been a surge of interest in immunotherapy as a novel adjunct to traditional cytotoxic oncologic therapies. With the clinical success of targeting checkpoint inhibitors CTLA4 and PD1 in melanoma, there is a broadened interest in applying immunotherapy to a larger spectrum of malignancies (45 trials combining aPD1 and RT, 35 trials combing aCTLA4 and RT [clinicaltrials.gov on 5/2/2016]). The reports of activity in non-melanoma cancers indicate it will become increasingly necessary to integrate immunotherapy alongside conventional therapies such as radiation or chemotherapy. However, while combinatorial use is becoming more prevalent, there are few studies designed to optimize therapeutic efficacy based on timing of administration of each agent alongside cytotoxic therapy. In this paper, we investigated the ideal timing of anti-CTLA4 blockade and anti-OX40 agonist therapy in combination with radiation, and found that optimal scheduling varied in accordance with the variable mechanisms of action of the immunotherapeutic agent.

Our results demonstrating that the optimal timing of anti-CTLA4 is before radiation therapy are consistent with anecdotal case reports from patients with metastatic melanoma receiving ipilimumab therapy where systemic responses with long-term disease-free survival occurs have been reported in patients who subsequently receive palliative radiation.[9, 10] Further, a retrospective review of patients receiving ipilumimab who underwent palliative radiation found improved overall survival if radiation was delivered during maintenance versus induction ipilumimab. While these data may be confounded by “healthier” patients avoiding palliative radiation long enough to receive radiation with maintenance ipilumimab versus induction, the results can also be interpreted to suggest that preconditioning with anti-CTLA4 improved outcome.[12] In murine models, concurrent and post-RT treatment with anti-CTLA4 has been shown to control tumor growth [6, 29], but had limited influence on overall survival, ranging from 0% [30] to 20% [31] overall survival with the combination of anti-CTLA4 and RT. The mechanism of action of anti-CTLA4 has been associated with its ability to deplete regulatory T cells in the tumor [19], and depletion of regulatory T cells concurrent or post-RT has been shown to improve tumor control by radiation therapy.[21, 22] Our data demonstrate that radiation followed by anti-CTLA4 blockade did improve radiation efficacy, but not to the same degree as pretreatment and that pretreatment depletion of regulatory T cells could also improve responses to radiation. These results are important given that the majority of ongoing clinical trials combining ipilimumab and radiation deliver ipilimumab concurrently and/or following radiation, which may result in improved outcomes, but may not be fully maximizing the potential for synergy. In one of the first clinical trial reports of clinical outcome of radiation therapy followed by ipilimumab, the combination resulted in 18% of patients demonstrating a partial response by RECIST criteria [32], which is not significantly different from the reported response of ipilimumab alone.[33] Our data were obtained using a relatively immunogenic tumor with high baseline Treg infiltrate which may influence ideal timing of anti-CTLA4 blockade. Additional tumor lines may demonstrate differences in timing based on differences in mechanisms of immune regulation. The MMTV-PyMT tumors in FVB mice demonstrated improved responses with immunotherapy, but this was not as effective as in seen with CT26, suggesting the mouse background and tumor type may influence overall survival. The baseline immunogenicity, tumor immune infiltrate and mouse background strain may influence efficacy of therapy and ideal timing of immune therapy with regards to radiation. In addition, our experiments were designed to identify the optimal timing of immune therapy relative to radiation and to determine if this varied based on the immunotherapeutic agent that was used. In order to avoid confounding factors of variations within the radiation dose or fractionation a single high dose treatment was chosen. Clinically, radiation dose and fractionation vary from a single high-dose treatment such as in early stage lung cancer and limited oligometastatic disease [34, 35] to low dose treatments delivered daily over weeks for both definitive and adjuvant treatment. As lower doses and or fractionated dose patterns may affect immune cell priming, trafficking and survival, loss of immunologic responses with radiation may be seen [36] and may confound questions of timing.

As opposed to the anti-CTLA4 checkpoint inhibitor, we found that anti-OX40 agonist antibodies, which act as T cell co-stimulatory agents, improve radiation efficacy when delivered shortly after radiation. The improved efficacy of combination therapy is consistent with the window of antigen presentation following hypofractionated radiation.[3] The OX40 molecule is upregulated on T cells rapidly and for a limited time following antigen engagement, and agonist antibodies must be present during that window for effective T cell stimulation.[17] While OX40 is expressed on regulatory T cells, administration of the OX86 anti-OX40 clone to tumor-bearing mice does not result in depletion of tumor regulatory T cells.[37] Anti-OX40 antibodies have recently shown promise in a phase I clinical trial at our institution [38], and are currently being evaluated in a Phase I trial in combination with radiation that uses the optimal timing described in this manuscript.

Additionally, these data demonstrate that using CT guidance, radiation therapy can be directed to spontaneous mammary tumors in mice, resulting in local control of invasive carcinoma. While we demonstrate that single-agent immunotherapy can extend survival in mice treated with radiation therapy, none of the mice were cured of these mammary tumors. In those mice that experience a benefit, there was a prolonged period of slower tumor growth, which has been described as an unbalanced equilibrium of tumor cell proliferation and immune control.[27, 28] Utilizing both anti-CTLA4 and anti-OX40 together with radiation we observed extended survival including a proportion of mice cured of their tumors. These data demonstrate that rationally combining different immunotherapies with cytotoxic therapy can improve outcomes. These data are closely related to those shown in a B16 melanoma mouse model where the combination of anti-CTLA4 with anti-PD1 and radiation therapy improved tumor control through non-redundant mechanisms.[32] In immunogenic tumors that achieve an equilibrium phase following radiation therapy, blocking PD1-PDL1 interactions results in tumor cures [27] and blocking PD1-PDL1 interactions can significantly extend survival in less immunogenic tumors even in protected environments.[39] In view of the increasing clinical data along with the extensive preclinical mouse data, it is likely that blocking PD1-PDL1 interactions would provide additional benefit in our model.

In conclusion, we demonstrate that the timing of immunotherapy in combination with radiation significantly affects outcome and that the ideal timing of specific immunotherapeutic agents depends on their mechanisms of action. We demonstrate that CT-guided radiation therapy permits accurate treatment of transplanted spontaneous transgenic mammary tumors in immune competent mice. The focal targeting allows modeling of the effect of tumor treatment on systemic immune responses and establishes a setting to test immunotherapies in this difficult-to-treat model of mammary tumor progression. Preclinical data using appropriate models addressing mechanism of action should be considered when combining agents and translating to the clinic. The cost and effort of clinical trials is such that optimizing the protocol for the most successful outcome should include these considerations.
